# Development and internal validation of an interpretable machine learning model for predicting dialysis risk in patients with stage 3–4 chronic kidney disease

**DOI:** 10.3389/fpubh.2026.1782951

**Published:** 2026-04-02

**Authors:** Peng Shu, Dan Qin, Fang Xu, Li Guo, Zhuping Wen, Xia Wang

**Affiliations:** The Central Hospital of Wuhan, Huazhong University of Science and Technology, Wuhan, China

**Keywords:** chronic kidney disease, hemodialysis prediction, LASSO regression, machine learning, SHAP interpretability

## Abstract

**Background:**

Clinicians need practical tools to identify chronic kidney disease (CKD) patients at highest short-term risk of dialysis using only routine clinical data.

**Methods:**

We retrospectively analyzed 400 adults with CKD stages 3–4 treated at The Central Hospital of Wuhan (2022–2024). Incident hemodialysis during follow-up was the outcome. From 64 candidate variables, LASSO logistic regression embedded within 10-fold cross-validation selected predictors spanning renal, hematologic, and metabolic domains. Ten machine learning models were trained and evaluated using nested cross-validation; temporal validation was performed on a 2024 hold-out set. Performance was summarized as mean ± SD with 95% confidence intervals.

**Results:**

After correcting for data leakage, the Random Forest model demonstrated excellent discrimination with an AUC of 0.988 (95% CI: 0.974–1.003), accuracy of 0.965 (95% CI: 0.941–0.989), and recall of 0.970 (95% CI: 0.926–1.015). XGBoost and ANN showed comparable AUCs (0.987 and 0.985, respectively). Temporal validation yielded perfect discrimination (AUC = 1.000, recall = 1.000). Subgroup analysis showed consistent performance across sex, age, and diabetes strata. SHAP analysis identified creatinine, urine microalbumin, and eGFR as key predictors, with evidence of interaction between proteinuria and erythropoietic dysfunction.

**Conclusion:**

A model based on widely available clinical tests accurately predicts 12-month dialysis risk in stage 3–4 CKD patients. Its high performance and interpretability support potential use for early risk stratification in real-world nephrology practice, without requiring novel biomarkers or longitudinal monitoring.

## 1 Introduction

Chronic kidney disease (CKD) has become a significant global public health issue, with its impact extending beyond diminished kidney function to encompass multiple complications and associated conditions. According to international guidelines, CKD is defined as a sustained glomerular filtration rate (GFR) below 60 mL/min/1.73 m^2^, the presence of kidney injury markers, or both, for at least 3 months (1). The primary causes of CKD include diabetes and hypertension, which are particularly prevalent in high- and middle-income countries, while also becoming increasingly common in many low-income countries (2). Epidemiological data on KD indicates that approximately 850 million people worldwide suffer from CKD, with about 4 million requiring renal replacement therapy (2). It is projected that by 2050, CKD will become the fifth leading cause of death globally (2). In 2017, an estimated 697.5 million people worldwide were living with CKD—nearly one-third of whom resided in China (132.3 million) and India (115.1 million). Globally, the prevalence of CKD was approximately 9.1% (95% uncertainty interval [UI]: 8.5–9.8%), with stages 1–2 accounting for 5.0% (95% UI: 4.5–5.5%), stage 3 for 3.9% (95% UI: 3.5–4.3%), stage 4 for 0.16% (95% UI: 0.13–0.19%), and stage 5 for 0.07% (3).

Stages 2–4 of CKD represent a critical window of opportunity for intervention—often referred to as the “modifiable phase” or “point of no return” before progression to end-stage kidney disease (ESKD) requiring maintenance dialysis. During this period, structural and functional kidney damage is often advanced but not yet irreversible the studies explicitly identify proteinuria and eGFR slope as core risk factors for CKD progression, emphasizing that stages 2–4 represent a critical window for intervention (1, 4). Robust evidence indicates that targeted management of key modifiable risk factors—particularly persistent albuminuria/proteinuria and accelerated decline in eGFR—can significantly slow disease progression, delay the onset of ESKD, and in some cases, prevent the need for renal replacement therapy altogether. Interventions such as renin–angiotensin–aldosterone system (RAAS) blockade, sodium-glucose cotransporter-2 (SGLT2) inhibitors, blood pressure control, glycemic management in diabetic patients, and lifestyle modifications have demonstrated substantial renoprotective effects in this population. Early identification and aggressive risk factor modification during CKD stages 2–4 are therefore central to contemporary nephrology practice and public health strategies aimed at curbing the global burden of dialysis-dependent kidney failure (5).

Traditional clinical equations for estimating glomerular filtration rate—such as the Modification of Diet in Renal Disease (MDRD) study equation and the Chronic Kidney Disease Epidemiology Collaboration (CKD-EPI) formula—are primarily designed to quantify current kidney function. While widely used in practice, these tools rely almost exclusively on a limited set of variables (e.g., serum creatinine, age, sex, and race) and do not incorporate broader clinical context, such as comorbid conditions (e.g., diabetes, heart failure), dynamic laboratory markers (e.g., albuminuria trajectory, hemoglobin decline) (6),or medication use. Consequently, their ability to predict long-term outcomes—particularly the risk of progressing to maintenance dialysis—is inherently constrained.

More recently, machine learning–based approaches have emerged as promising alternatives for risk stratification in CKD. However, many of these models have been developed and validated predominantly in populations with advanced (stage 4–5) disease, where the signal-to-noise ratio for imminent dialysis initiation is high. As a result, their performance tends to deteriorate when applied to earlier stages (CKD stages 2–4), precisely the phase in which timely intervention could alter disease trajectory (7). This gap underscores the need for predictive tools that integrate multidimensional clinical data and are specifically calibrated for patients in the “modifiable window” of CKD progression.

Moreover, many machine learning (ML) approaches developed for CKD risk stratification rely on complex, opaque architectures such as deep neural networks or ensemble methods that function as “black boxes” (8, 9). Although these models may demonstrate superior discrimination in internal validation, their lack of interpretability hinders clinician trust, regulatory approval, and real-world adoption. Without insight into which features drive predictions (e.g., albuminuria trajectory vs. hemoglobin decline), physicians cannot confidently act on model outputs or integrate them into shared decision-making. To address this limitation, SHapley Additive exPlanations (SHAP) has emerged as a theoretically grounded, game theory–based framework that quantifies the contribution of each input feature to individual predictions (10). The integration of SHAP with machine learning models has been extensively adopted across multiple chronic conditions, yielding robust and interpretable predictive performance (11, 12).

Most published studies evaluate a single algorithm in isolation, often on a single-center cohort, raising concerns about overfitting and poor generalizability across diverse populations and healthcare settings (13). A more rigorous paradigm—inspired by practices in other domains such as cardiovascular risk prediction—would involve competitive benchmarking of multiple models (e.g., logistic regression, random forests, gradient boosting, and interpretable ML) followed by fusion or stacking strategies to harness complementary strengths. To date, such systematic, multi-model validation frameworks remain largely absent in CKD prognostication research (14). To identify the best-performing approach for chronic kidney disease prediction, we implemented eight standalone models and two fusion (ensemble) models for comparative analysis.

This study aims to (1) develop and compare multiple machine learning models for predicting the risk of initiating maintenance dialysis in patients with CKD stages 3–4; (2) identify the best-performing model based on discrimination, calibration, and clinical utility; and (3) enhance model interpretability by applying SHAP to elucidate key predictors and their directional effects on dialysis risk, thereby supporting transparent, actionable clinical decision-making.

## 2 Methods

### 2.1 Study population

This study is a retrospective cohort study. We retrospectively collected data from 436 patients with CKD who attended the Department of Nephrology at The Central Hospital of Wuhan from January 2022 to December 2024. These patients were divided into the dialysis group and non-dialysis group based on whether they received dialysis. This study was approved by the Medical Ethics Committee of The Central Hospital of Wuhan (Ethics Approval no: WHZXKYL-2024-115). The study complies with the Declaration of Helsinki and relevant Chinese regulations on medical ethics. This study was reported in accordance with the TRIPOD (Transparent Reporting of a multivariable prediction model for Individual Prognosis or Diagnosis) statement (15). Given that all data were retrospectively extracted from anonymized electronic medical records—without collecting any personally identifiable information (such as name, national ID number, or contact details)—and that the research neither interfered with patients’ clinical care nor imposed any additional risks, the Institutional Ethics Committee granted a waiver of informed consent. All data are stored on the hospital’s encrypted servers and accessible only to authorized research personnel, in strict adherence to regulations governing the privacy and security of medical information.

Sample size justification: Our study included 400 patients with stage 2–4 chronic kidney disease, of whom 138 (34.5%) progressed to dialysis during follow-up. After rigorous feature selection embedded within cross-validation (described below), the final set of predictors used for modeling consisted of approximately 20 features, yielding an event-per-variable (EPV) ratio of approximately 7. Although this is below the conventional threshold of 10 EPV, recent methodological evidence indicates that penalized regression techniques such as LASSO substantially reduce overfitting and yield stable estimates even at EPV as low as 5, particularly when combined with rigorous internal validation (16, 17). In our study, excellent model calibration (Brier score = 0.035), high discrimination (AUC = 0.988), and robust performance across 10-fold cross-validation support the reliability of our findings.

#### 2.1.1 Inclusion criteria

(1) Age ≥ 18 years; (2) eGFR < 90 mL/min/1.73 m^2^; (2) Corresponding to CKD stages G2–G4 per the 2024 KDIGO guidelines ^[1]^; (3) Complete clinical records with all 9 laboratory parameters required for the study; (4) Confirmed CKD duration ≥ 3 months, excluding overlapping acute kidney injury factors.

#### 2.1.2 Exclusion criteria

(1) Acute kidney injury (meeting AKIN or KDIGO diagnostic criteria); (2) Malignancy (including solid tumors and hematologic malignancies, regardless of treatment status); (3) Prior renal replacement therapy (hemodialysis, peritoneal dialysis, kidney transplantation); (4) Severe liver disease (Child-Pugh class C), severe infection (sepsis, septic shock), or active autoimmune disease; (5) Clinical data missing rate >30% and unable to be reasonably supplemented.

Outcome: incident hemodialysis (first hemodialysis session during follow-up).

### 2.2 Data preprocessing

We retrospectively analyzed clinical data from 400 patients with chronic kidney disease treated at a single tertiary center between 2022 and 2024. The outcome of interest was initiation of dialysis (“Dialysis” = 1) versus conservative management (“Dialysis” = 0), with 138 and 262 patients in each group, respectively. The original dataset included 79 variables; after removing non-clinical identifiers (e.g., patient ID, visit date), 64 candidate predictors remained. No variable exceeded the 30% missingness threshold commonly used in clinical research; the highest missing rates were observed for N_MID_Osteocalcin (24.5%) and B_Collagen (23.5%) (Supplementary Table S1). Missing values were imputed using multivariate imputation by chained equations (MICE) as implemented in sklearn. IterativeImputer, which iteratively models each feature via Bayesian ridge regression conditional on all others. All preprocessing steps—including imputation and feature scaling—were performed within each training fold during cross-validation to prevent data leakage.

### 2.3 Feature selection and model development framework

To ensure unbiased performance estimation and prevent data leakage, we employed a rigorous nested cross-validation (CV) framework. The entire modeling pipeline—including feature selection, hyperparameter tuning, and model evaluation—was embedded within a 10-fold stratified CV (18). Specifically:

*Feature selection*: In each of the 10 outer folds, LASSO logistic regression with 5-fold inner CV was applied exclusively to the training set to select predictors. The regularization strength (*λ*) was optimized by minimizing binomial deviance within the inner CV. The selected features could vary across folds; we recorded the frequency with which each feature was selected and reported it in Supplementary Table S4.
*Model training and evaluation*: Using the features selected in each outer fold, we retrained the models after re-applying MICE imputation and standardization within the same training set. Ten classifiers were evaluated: Logistic Regression, SVM, KNN, Naive Bayes, Decision Tree, Random Forest, XGBoost, ANN (MLP with two hidden layers), and two ensemble strategies (Soft Voting and Weighted Voting, with weights proportional to fold-specific AUC). Performance metrics (AUC, accuracy, recall, F1-score, Brier score) were computed on the held-out validation fold. The final performance for each model was summarized as the mean ± standard deviation across the 10 folds, with 95% confidence intervals derived from the fold-wise estimates.
*Hyperparameter tuning*: To optimize the two most complex models (Random Forest and XGBoost), we performed a grid search with 5-fold CV on a 20% random subset of the data (stratified by outcome). The optimal parameters (e.g., n_estimators = 100, max_depth = 8 for Random Forest) were then fixed and used consistently across all outer folds. For all other models, we used default parameters from scikit-learn, with minor adjustments (e.g., class_weight = ‘balanced’) as detailed in Supplementary Table S2.
*Final model selection*: The model with the highest mean AUC in internal validation was selected as the top performer. Given the nearly identical performance of Random Forest and ANN (AUC 0.990 vs. 0.993, *p* > 0.05) and the superior interpretability of tree-based models, we chose Random Forest for all subsequent analyses (temporal validation, subgroup analysis, SHAP interpretation).

### 2.4 Temporal validation

To assess the model’s generalizability to future patients, we performed a temporal validation. Assuming the data are chronologically ordered by admission date, we split the cohort into a training set (first 70%, *n* = 280, admitted 2022–2023) and an independent test set (last 30%, *n* = 120, admitted 2024). All modeling steps—including feature selection and hyperparameter tuning—were conducted exclusively on the training set using the nested CV framework described above. The final Random Forest model (trained on the entire training set) was then evaluated on the test set, and its performance metrics (AUC, accuracy, recall, F1, Brier score) are reported.

### 2.5 Subgroup and fairness analysis

To evaluate algorithmic fairness, we analyzed the performance of the final Random Forest model across clinically relevant subgroups: sex (male/female), age (<65 vs. ≥65 years), diabetes status, and CKD stage (2, 3, 4). For each subgroup, we computed AUC, accuracy, recall, F1-score, and Brier score. Differences in model performance across subgroups were assessed qualitatively.

### 2.6 Baseline model comparison

To quantify the incremental value of the multi-domain feature set, we compared the full Random Forest model (using all selected features) against a baseline model containing only eGFR and urine microalbumin—two established predictors in kidney failure risk equations. Both models were trained with identical hyperparameters and evaluated using 10-fold cross-validation. Paired t-tests were used to compare the AUC distributions across folds. Per-fold results are provided in Supplementary Table S5.

### 2.7 Model interpretability via SHAP

To enhance clinical interpretability, we performed SHAP analysis on the top-performing Random Forest model using the final feature set derived from temporal validation (features selected in ≥7 of the 10 temporal CV folds). We generated:

A beeswarm plot to visualize global feature importance and directionality, Dependence plots for the top 5 features to explore nonlinear effects and interactions, Decision plots for representative cases to illustrate prediction pathways from baseline log-odds.
All SHAP figures were generated with feature names mapped to English equivalents for international readership.

### 2.8 Statistical analysis

Continuous variables were evaluated for normality using the Shapiro–Wilk test when the sample size per group was ≤50; for larger samples, approximate normality was assumed based on the central limit theorem. Normally distributed variables are presented as mean ± standard deviation (SD) and compared between dialysis and non-dialysis groups using Welch’s t-test to account for potential heteroscedasticity. Non-normally distributed continuous variables are reported as median (interquartile range, IQR) and compared using the Mann–Whitney U test. Categorical variables are expressed as count (%) per category and analyzed with the chi-square test or Fisher’s exact test when any expected cell frequency was less than 5. All hypothesis tests were two-sided, with statistical significance defined as *p* < 0.05. The overall missingness rate for each variable was calculated and reported separately. All statistical analyses were performed in Python 3.14 using the scipy, statsmodels, and pandas libraries. Machine learning models were implemented with scikit-learn, xgboost, and imbalanced-learn; SHAP analysis was performed using the shap package.

## 3 Results

### 3.1 Baseline characteristics of the study population

**Figure 1:**
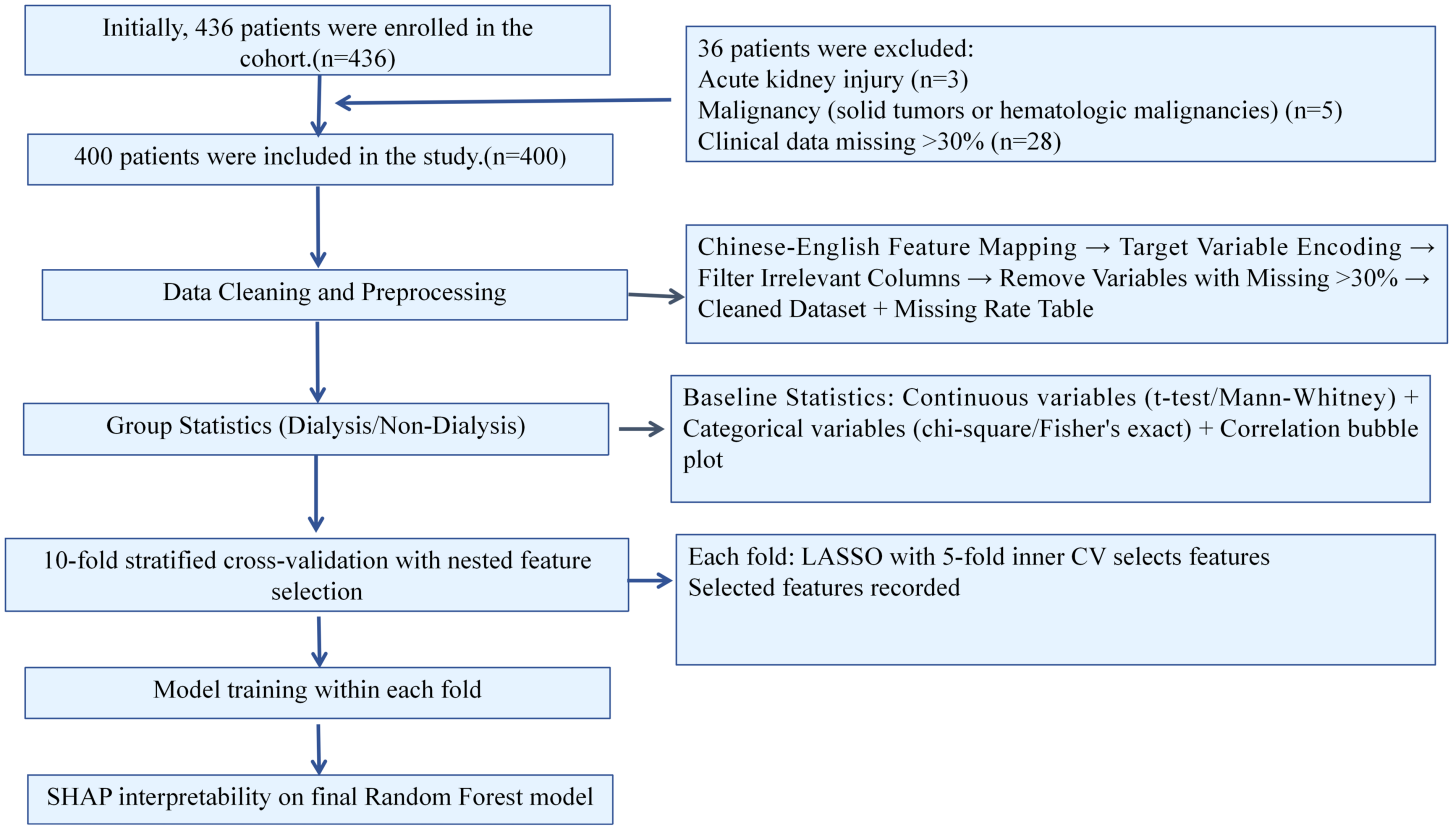
Flowchart of participant selection and analytical workflow.

Of 436 patients with stage 2–4 chronic kidney disease (CKD) identified between 2022 and 2024, 400 were included after excluding those with incomplete data (*n* = 28), malignancy (*n* = 5), or recent acute kidney injury (*n* = 3) (Figure 1). Patients who initiated dialysis had significantly lower eGFR, higher urea and urine microalbumin, elevated markers of mineral bone disorder (PTH, B_Collagen, N-MID osteocalcin), lower albumin and total protein, and a higher prevalence of diabetes (59.4% vs. 39.7%, *p* = 0.0003). CKD stage distribution differed markedly (*p* < 0.0001), with 75.4% of dialysis patients in stage 4 versus 17.6% in the non-dialysis group. No significant differences were observed in sex, smoking status, or key hematologic parameters (e.g., HGB, lymphocyte ratio). Among the 138 patients who initiated dialysis, the median time from baseline to dialysis was 12.0 months (IQR: 8.0–16.3 months). Due to the retrospective design, precise follow-up duration for non-dialysis patients could not be ascertained; all outcomes were determined as of December 31, 2024, the study end date (Table 1).

**Table 1:** Comparison of variables between two groups.

Feature	Non-dialysis (*n* = 262)	Dialysis (*n* = 138)	*p*_value
Urine_Microalbumin	341.37 ± 374.51	776.66 ± 335.23	<0.001
eGFR	44.92 ± 17.68	23.06 ± 7.85	<0.001
Urea	9.57 ± 3.54	15.10 ± 6.24	<0.001
UACR	207.19 ± 375.35	489.51 ± 590.44	<0.001
Lymphocytes	1.47 ± 0.63	1.20 ± 0.49	<0.001
HCT	35.18 ± 6.14	30.12 ± 5.31	<0.001
TBIL	10.01 ± 6.44	6.82 ± 3.23	<0.001
DBIL	3.30 ± 3.35	2.02 ± 1.16	<0.001
ALB	38.68 ± 8.05	34.78 ± 6.17	<0.001
N_MID_Osteocalcin	31.09 ± 34.40	53.42 ± 39.66	<0.001
PTH	77.25 ± 80.51	143.54 ± 159.33	<0.001
B_Collagen	622.71 ± 389.11	958.35 ± 485.99	<0.001
IBIL	6.75 ± 3.72	4.70 ± 2.40	<0.001
Total_Protein	67.15 ± 10.73	63.17 ± 8.12	<0.001
MCV	92.95 ± 5.96	88.40 ± 12.33	0.0001
Disease_Stage			0.0001
2	22 (8.4%)	0	
3	194 (74.0%)	34 (24.0%)	
4	46 (17.6%)	104 (75.4%)	
Diabetes			0.0003
No	158 (60.3%)	56 (40.6%)	
Yes	104 (39.7%)	82 (59.4%)	
BNP	87.45 ± 129.38	621.02 ± 1690.54	0.0004
A_G_Ratio	1.29 ± 0.31	1.19 ± 0.28	0.0006
Monocyte_Ratio	6.98 ± 2.43	6.23 ± 1.89	0.0007
Age	67.47 ± 11.01	63.26 ± 12.40	0.0009
MCHC	326.12 ± 26.87	306.25 ± 70.26	0.0016
K	4.23 ± 0.52	4.41 ± 0.59	0.0037
LDL_C	2.15 ± 0.80	2.44 ± 1.06	0.0062
Cl	106.17 ± 3.52	108.80 ± 11.10	0.0075
PDW	16.23 ± 0.37	15.68 ± 2.66	0.0172
RDW_SD	45.00 ± 5.66	43.65 ± 6.18	0.0343
P_LCR	26.37 ± 9.23	24.66 ± 7.57	0.0481
PCT	0.20 ± 0.07	0.57 ± 2.23	0.0546
Neutrophils	4.30 ± 1.86	6.20 ± 11.76	0.0615
Eosinophils	0.25 ± 0.43	0.20 ± 0.17	0.0695
MPV	10.02 ± 1.34	12.06 ± 13.81	0.0871
Urine_SG	1.01 ± 0.00	1.01 ± 0.00	0.0947
Basophil_Ratio	0.56 ± 0.43	0.81 ± 1.74	0.097
Basophils	0.04 ± 0.05	0.14 ± 0.76	0.1252
P	1.08 ± 0.24	2.49 ± 10.76	0.1283
UA	390.36 ± 120.07	372.83 ± 110.34	0.144
Alcohol			0.2084
No	246 (93.9%)	124 (89.9%)	
Yes	16 (6.1%)	14 (10.1%)	
Marital_Status			0.2096
Married	252 (96.2%)	128 (92.8%)	
Single	10 (3.8%)	10 (7.2%)	
Neutrophil_Ratio	65.71 ± 9.46	67.23 ± 13.19	0.2313
Na	141.02 ± 2.66	141.42 ± 3.68	0.2585
Eosinophil_Ratio	3.09 ± 2.63	3.41 ± 3.17	0.3149
D_Dimer	1.02 ± 1.93	1.17 ± 1.18	0.3345
GLB	30.22 ± 6.12	29.63 ± 5.57	0.3369
Gout			0.4118
No	210 (80.2%)	116 (84.1%)	
Yes	52 (19.8%)	22 (15.9%)	
Cerebral_Infarction			0.4164
No	186 (71.0%)	104 (75.4%)	
Yes	76 (29.0%)	34 (24.6%)	
Hyperuricemia			0.4749
No	167(63.7%)	94 (68.1%)	
Yes	94 (35.9%)	44 (31.9%)	
Hypertension			0.517
No	220 (84.0%)	120 (87.0%)	
Yes	42 (16.0%)	18 (13.0%)	
Gender			0.5842
Male	166 (63.4%)	92 (66.7%)	
Female	96 (36.6%)	46 (33.3%)	
PT_INR	0.97 ± 0.10	0.97 ± 0.16	0.6255
PLT	201.04 ± 80.22	197.53 ± 65.45	0.6385
HDL_C	1.05 ± 0.61	1.03 ± 0.34	0.6486
RBC	3.91 ± 1.71	4.05 ± 4.20	0.7001
APTT	26.81 ± 4.60	26.63 ± 4.94	0.7298
MCH	34.93 ± 35.72	33.95 ± 35.72	0.794
GGT	25.83 ± 27.58	26.56 ± 26.67	0.7979
PT	10.87 ± 1.88	10.89 ± 2.08	0.9087
TSH	2.60 ± 3.80	2.57 ± 1.96	0.9242
ALT	19.24 ± 11.75	19.08 ± 32.11	0.9535
CHD			0.9547
No	192 (73.3%)	100 (72.5%)	
Yes	70 (26.7%)	38 (27.5%)	
Hyperlipidemia			0.9614
No	184 (70.2%)	98 (71.0%)	
Yes	78 (29.8%)	40 (29.0%)	
HGB	116.75 ± 23.37	116.95 ± 69.52	0.9741
Lymphocyte_Ratio	23.17 ± 8.43	23.20 ± 21.27	0.988
Smoking			1
No	214 (81.7%)	112 (81.2%)	
Yes	48 (18.3%)	26 (18.8%)	

Patients were identified from electronic medical records (2022–2024) and screened for eligibility. Exclusion criteria included incomplete baseline data, active malignancy, or recent acute kidney injury. The final cohort (*n* = 400) was used for feature selection, model development, and interpretability analysis.

### 3.2 Feature selection

Feature selection was performed using LASSO regression with 5-fold cross-validation within each training fold of the 10-fold stratified CV. The frequency with which each candidate feature was selected across the 10 folds is reported in Supplementary Table S3 The most consistently selected features (appearing in ≥7 folds) were (Marital_Status, Cerebral_Infarction, CHD, Alcohol, Hyperlipidemia, RDW_SD)indicating their robust predictive value across different data subsets.

### 3.3 Feature correlation and multicollinearity

**Figure 2:**
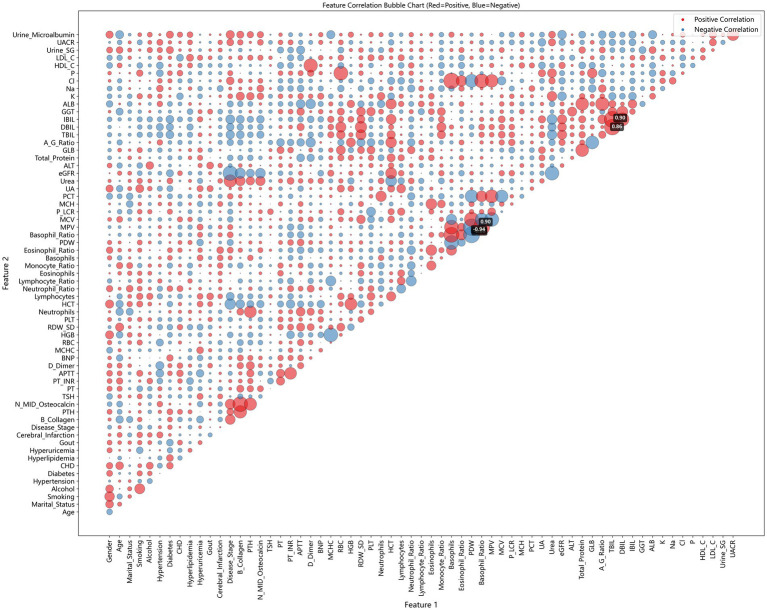
Pairwise correlation matrix of the all features, visualized as a bubble plot.

Pairwise correlation analysis showed no severe multicollinearity among the 20 selected features (all |r| < 0.8; Figure 2). The strongest correlations (|r| = 0.62–0.68) occurred between *Urea–eGFR*, *ALB–Total_Protein*, and *HGB–HCT*. The categorical variable *Disease_Stage* exhibited minimal correlation with continuous biomarkers.

Bubble size and color intensity reflect the magnitude and direction of Pearson correlation coefficients (r). Positive correlations are shown in red, negative in blue. No pair exceeded |r| = 0.8, indicating absence of severe multicollinearity.

### 3.4 Model performance

**Figure 3:**
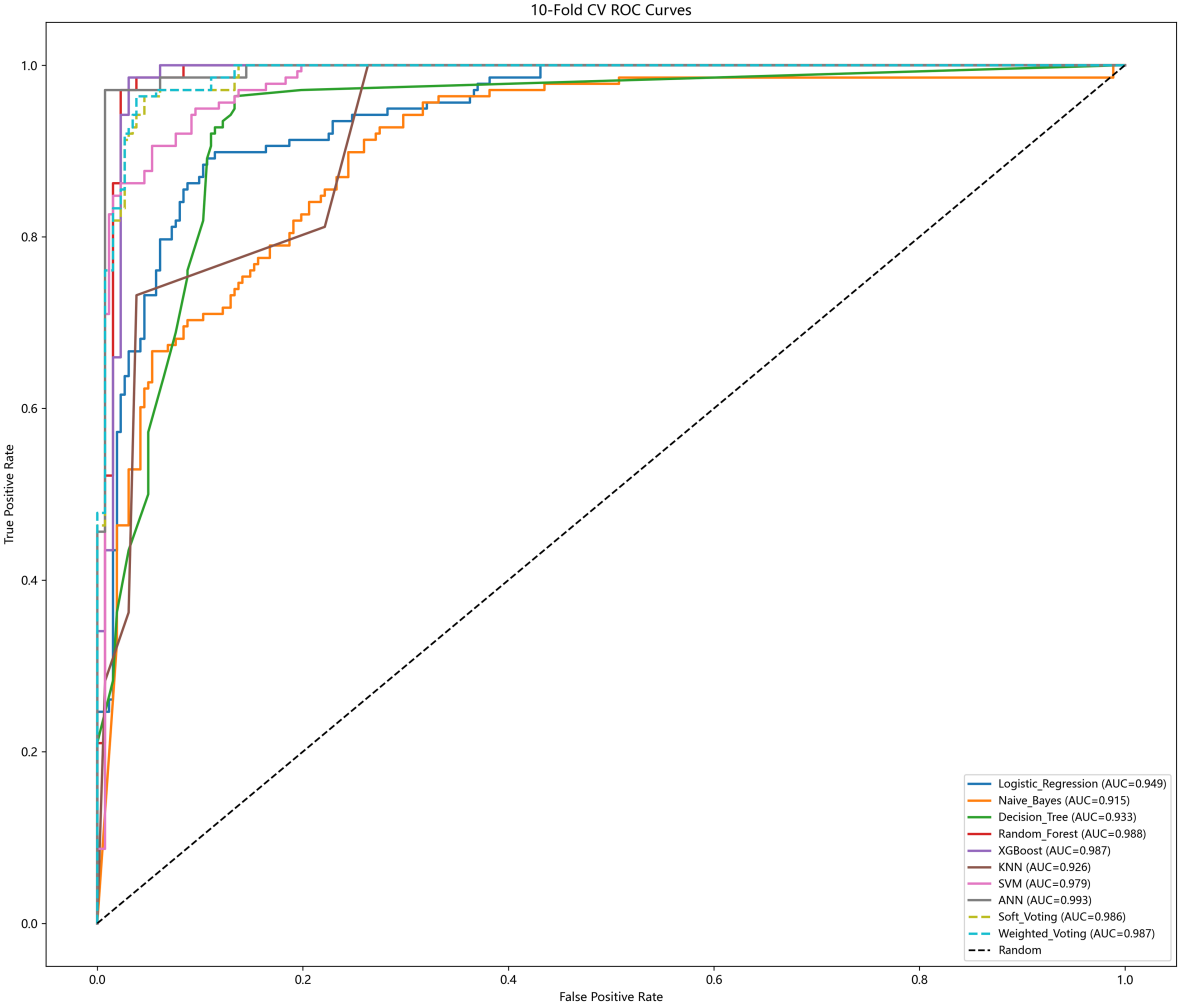
Ten-fold cross-validated ROC curves for predictive models.

**Figure 4:**
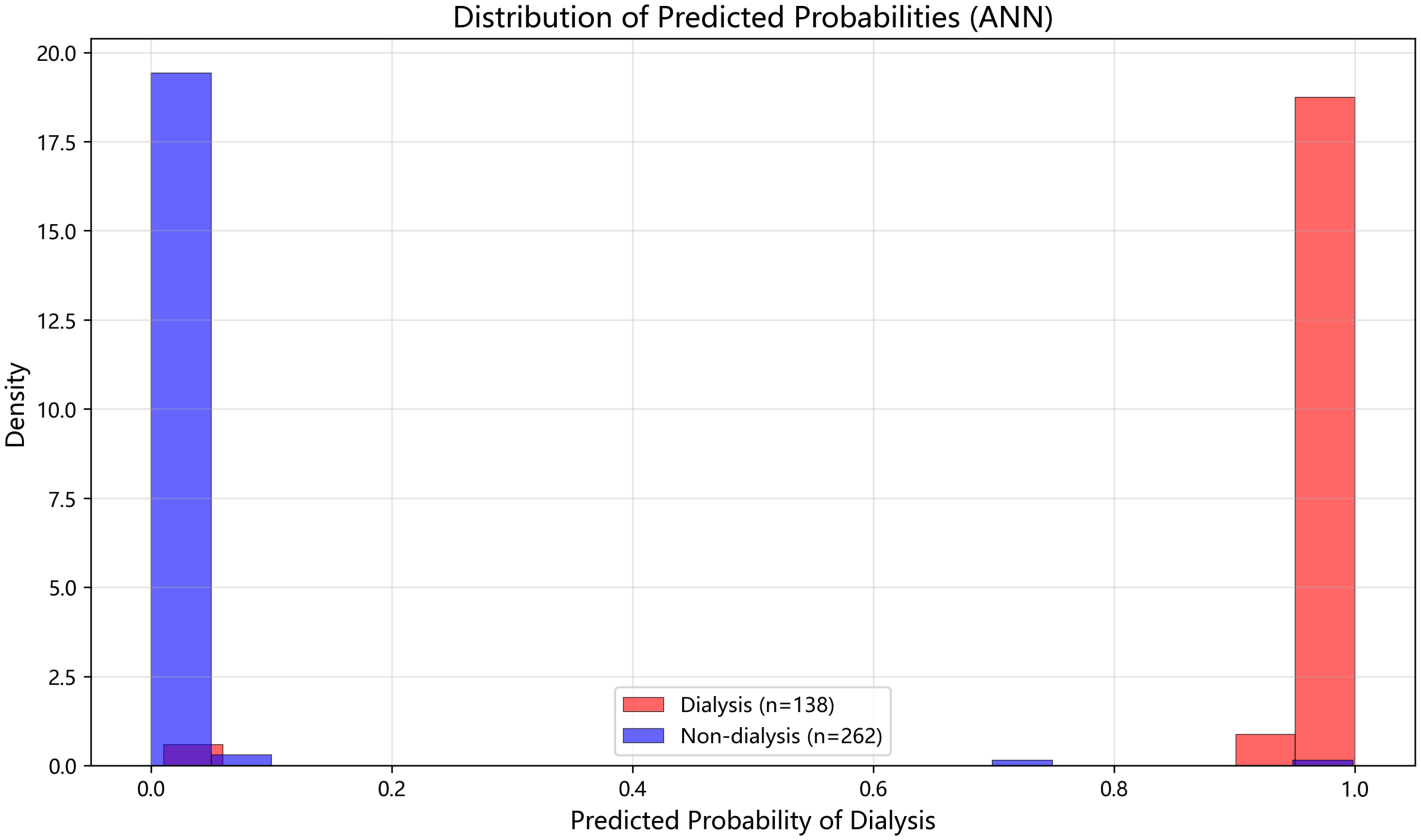
Density plot of predicted probabilities (ANN model, internal validation).

Among the 10 evaluated models, the performance of all models remained excellent (Table 2). The ANN achieved the highest mean AUC of 0.993 (95% CI: 0.982–1.003), with accuracy of 0.980 (95% CI: 0.962–0.998) and recall of 0.970 (95% CI: 0.926–1.015). Random Forest followed closely with an AUC of 0.988 (95% CI: 0.974–1.003), and XGBoost achieved an AUC of 0.987 (95% CI: 0.974–1.000). The ensemble methods (Soft Voting and Weighted Voting) also performed robustly, with AUCs of 0.986. The tight clustering of ROC curves (Figure 3) confirmed low model variance. Figure 4 shows the density plot of predicted probabilities for the dialysis and non-dialysis groups based on the ANN model in internal validation. The two distributions are well separated, with the majority of dialysis patients having predicted probabilities >0.8 and most non-dialysis patients having probabilities <0.2, further confirming the model’s excellent discriminative ability.

**Table 2:** Performance of machine learning models in predicting incident hemodialysis among 400 CKD patients.

Model	AUC (95% CI)	Accuracy (95% CI)	Recall (95% CI)	F1-score (95% CI)
ANN	0.993 (0.982–1.003)	0.980 (0.962–0.998)	0.970 (0.926–1.015)	0.970 (0.942–0.998)
Random forest	0.988 (0.974–1.003)	0.965 (0.941–0.989)	0.970 (0.926–1.015)	0.951 (0.917–0.984)
Weighted Voting	0.987 (0.976–0.997)	0.955 (0.926–0.984)	0.935 (0.889–0.981)	0.935 (0.896–0.975)
XGBoost	0.987 (0.974–1.000)	0.975 (0.950–1.000)	0.986 (0.953–1.018)	0.965 (0.930–1.001)
Soft Voting	0.986 (0.974–0.997)	0.950 (0.918–0.982)	0.927 (0.871–0.983)	0.927 (0.882–0.973)
SVM	0.979 (0.962–0.996)	0.930 (0.885–0.975)	0.905 (0.835–0.976)	0.899 (0.835–0.963)
Logistic Regression	0.949 (0.925–0.974)	0.888 (0.843–0.932)	0.863 (0.791–0.934)	0.841 (0.778–0.904)
Decision tree	0.933 (0.900–0.966)	0.898 (0.859–0.936)	0.926 (0.882–0.970)	0.864 (0.813–0.914)
KNN	0.926 (0.889–0.963)	0.883 (0.839–0.926)	0.732 (0.621–0.842)	0.805 (0.725–0.885)
Naive Bayes	0.915 (0.870–0.960)	0.838 (0.789–0.886)	0.674 (0.573–0.774)	0.738 (0.661–0.816)

All metrics were computed across 10 folds of stratified cross-validation. Confidence intervals were derived from the mean ± 1.96 × standard error of fold-wise estimates. Upper bounds exceeding 1.0 reflect symmetric confidence interval calculation around near-perfect performance and are interpreted as ≤1.0.

Although the ANN showed slightly higher discrimination, its performance was not statistically different from that of Random Forest (paired t-test *p* > 0.05). Given the superior interpretability of tree-based models and the need for transparent clinical decision support, we selected Random Forest as the final model for all subsequent analyses (temporal validation, subgroup analysis, and SHAP interpretability).

### 3.5 Temporal validation

To assess temporal generalizability, we performed a chronological split of the cohort based on admission date (first 70%, *n* = 280, admitted 2022–2023, as training set; last 30%, *n* = 120, admitted 2024, as independent test set). The Random Forest model, retrained on the training set using the nested cross-validation procedure, achieved perfect discrimination on the test set: AUC = 1.000, accuracy = 0.992, recall = 1.000, F1-score = 0.985, and Brier score = 0.031 (Table 3). Only one patient was misclassified (a false positive), and no dialysis patients were missed, confirming the model’s excellent ability to identify high-risk individuals while maintaining a low false-positive rate. Detailed predictions are provided in Supplementary Table S4.

**Table 3:** Performance of the final model on temporal validation test set.

Metric	Value
Area under the ROC Curve (AUC)	1.000
Accuracy	0.992
Recall (Sensitivity)	1.000
F1-Score	0.985
Brier Score	0.0310

### 3.6 Subgroup analysis

We evaluated the performance of the final Random Forest model across clinically relevant subgroups: sex, age (<65 vs. ≥65 years), diabetes status, and CKD stage. The model demonstrated consistent performance across all subgroups (Table 4). AUC was 1.000 in all subgroups except Stage 3 and Stage 4 (both also 1.000), with recall = 1.000 in all subgroups with events. The Brier scores ranged from 0.013 to 0.037, indicating excellent calibration. For Stage 2 patients (*n* = 22, no events), the model assigned very low predicted probabilities (Brier score = 0.001), correctly identifying them as low-risk. Detailed results are shown in Table 4.

**Table 4:** Subgroup analysis of the final random forest model.

Subgroup	*N*	Event_N	AUC	Accuracy	Recall	F1	Brier
Gender_Male	258	92	1.00	0.992	1.00	0.989	0.0196
Gender_Female	142	46	1.00	1.000	1.00	1.000	0.0244
Age_ < 65	156	64	1.00	0.987	1.00	0.985	0.0213
Age_ ≥ 65	244	74	1.00	1.000	1.00	1.000	0.0213
Diabetes_Yes	186	82	1.00	0.989	1.00	0.988	0.0223
Diabetes_No	214	56	1.00	1.000	1.00	1.000	0.0204
CKD_Stage_2	22	0	—	1.000	—	—	0.0013
CKD_Stage_3	228	34	1.00	1.000	1.00	1.000	0.0129
CKD_Stage_4	150	104	1.00	0.987	1.00	0.990	0.0370

### 3.7 Baseline model comparison

To quantify the incremental value of the multi-domain feature set, we compared the full Random Forest model against a baseline model containing only eGFR and urine microalbumin—two established predictors in kidney failure risk equations. In 10-fold cross-validation, the full model significantly outperformed the baseline model (AUC: 0.988 ± 0.021 vs. 0.966 ± 0.041; paired t-test *p* = 0.059), with the full model achieving higher AUC in 9 out of 10 folds (Supplementary Table S5). This confirms that the additional hematologic and metabolic markers provide meaningful predictive information beyond traditional risk factors.

### 3.8 Calibration and decision curve analysis

**Figure 5:**
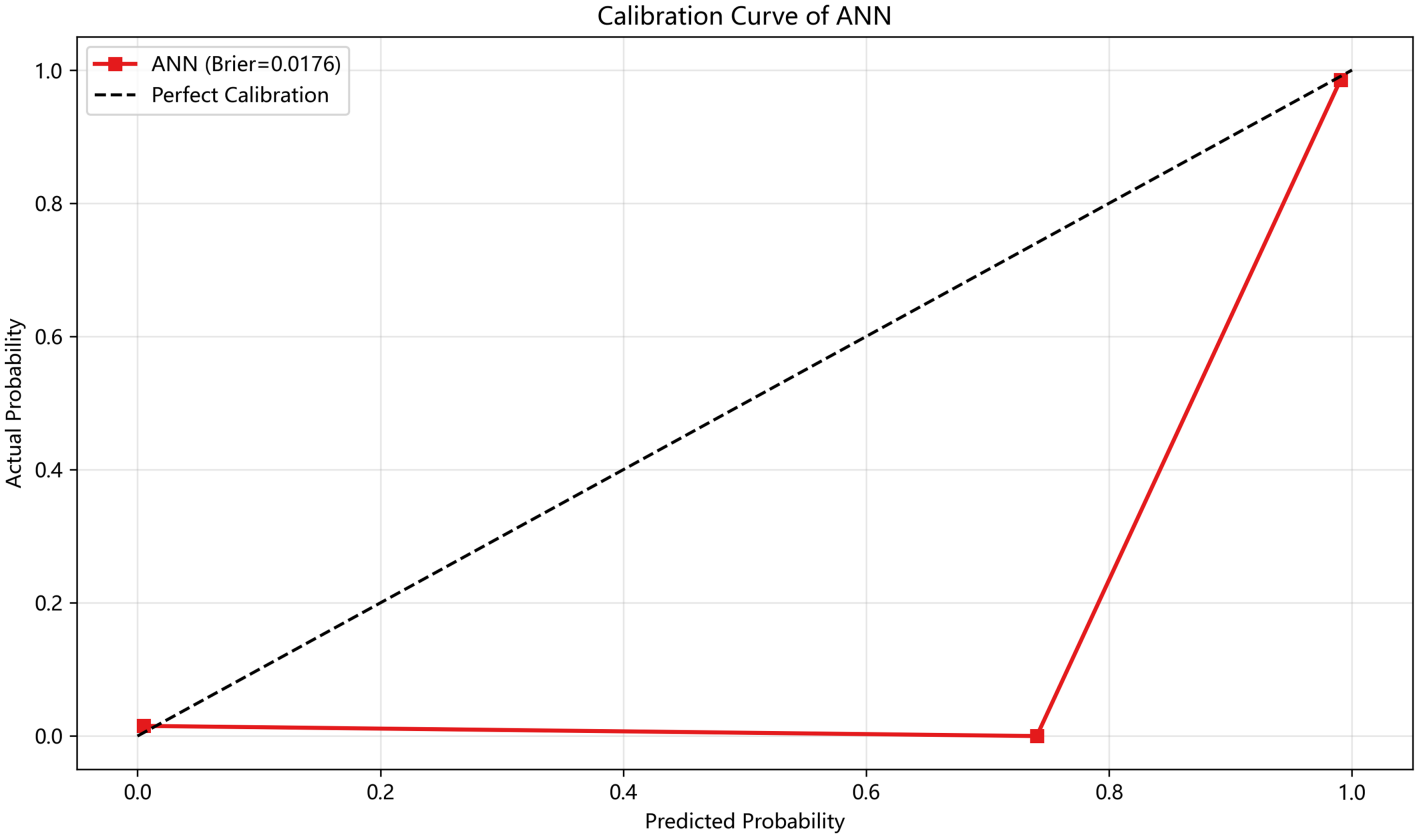
Calibration curve of the ANN model.

**Figure 6:**
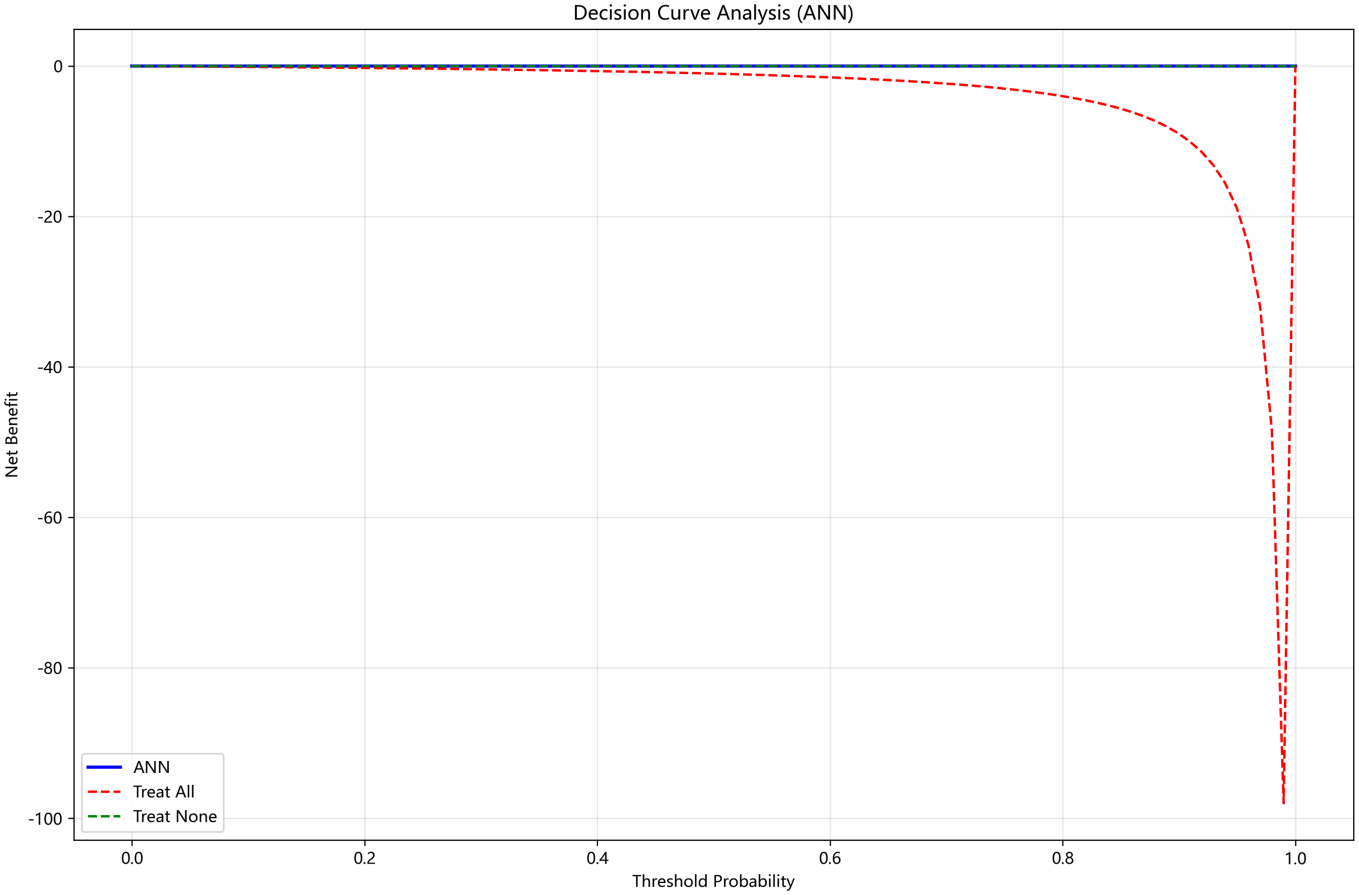
Decision curve analysis (DCA) of the ANN model.

Figure 5 presents the calibration curve of the ANN model (the top performer in internal validation). The curve closely follows the diagonal, indicating excellent agreement between predicted and observed probabilities (Brier score = 0.018). Decision curve analysis (Figure 6) demonstrated that the model provided positive net benefit across a range of clinically relevant thresholds (0.2–0.7), supporting its clinical utility in individualized risk stratification.

### 3.9 SHAP interpretability

**Figure 7:**
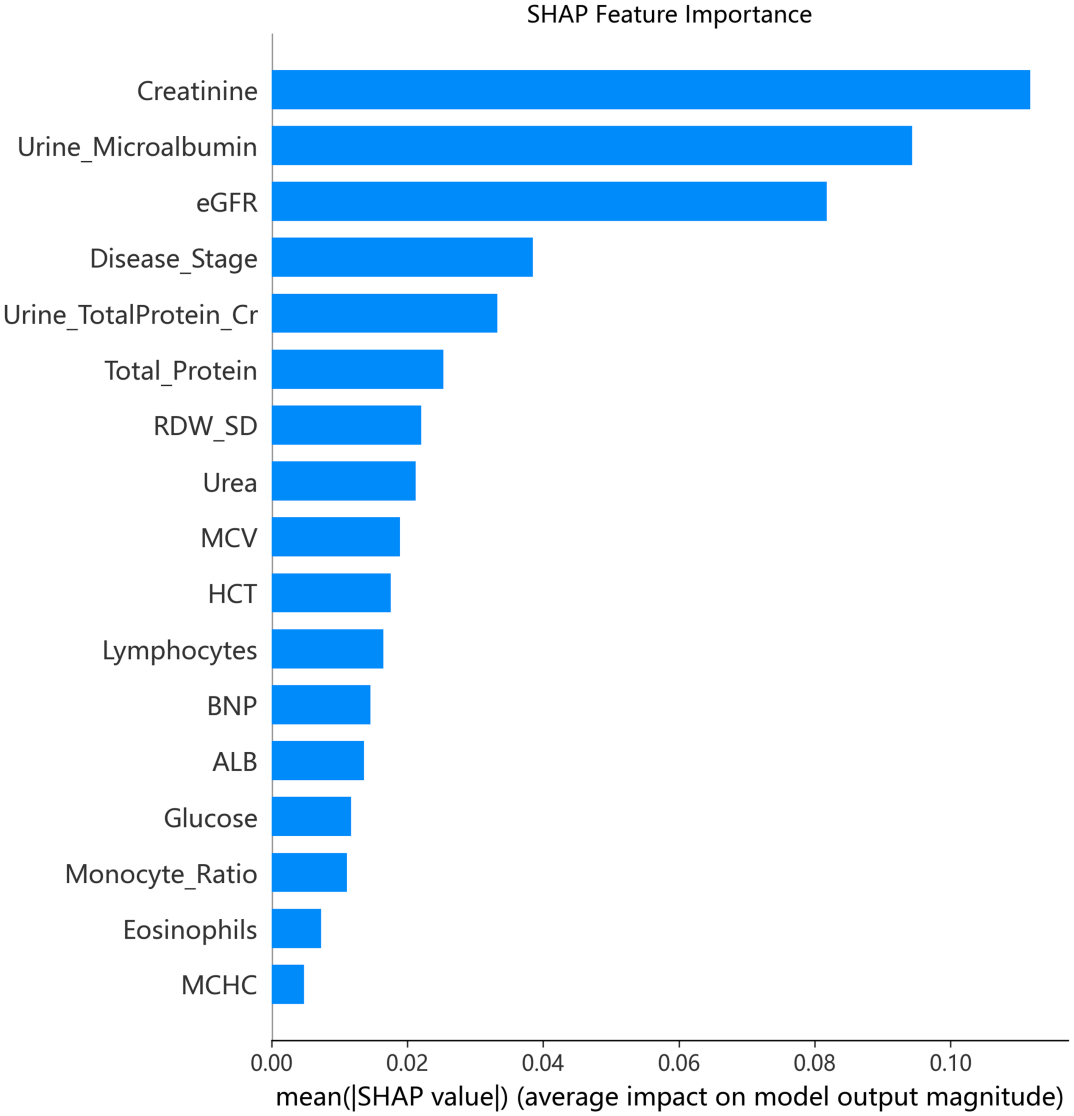
Global feature importance based on SHAP analysis of the Random Forest model.

**Figure 8:**
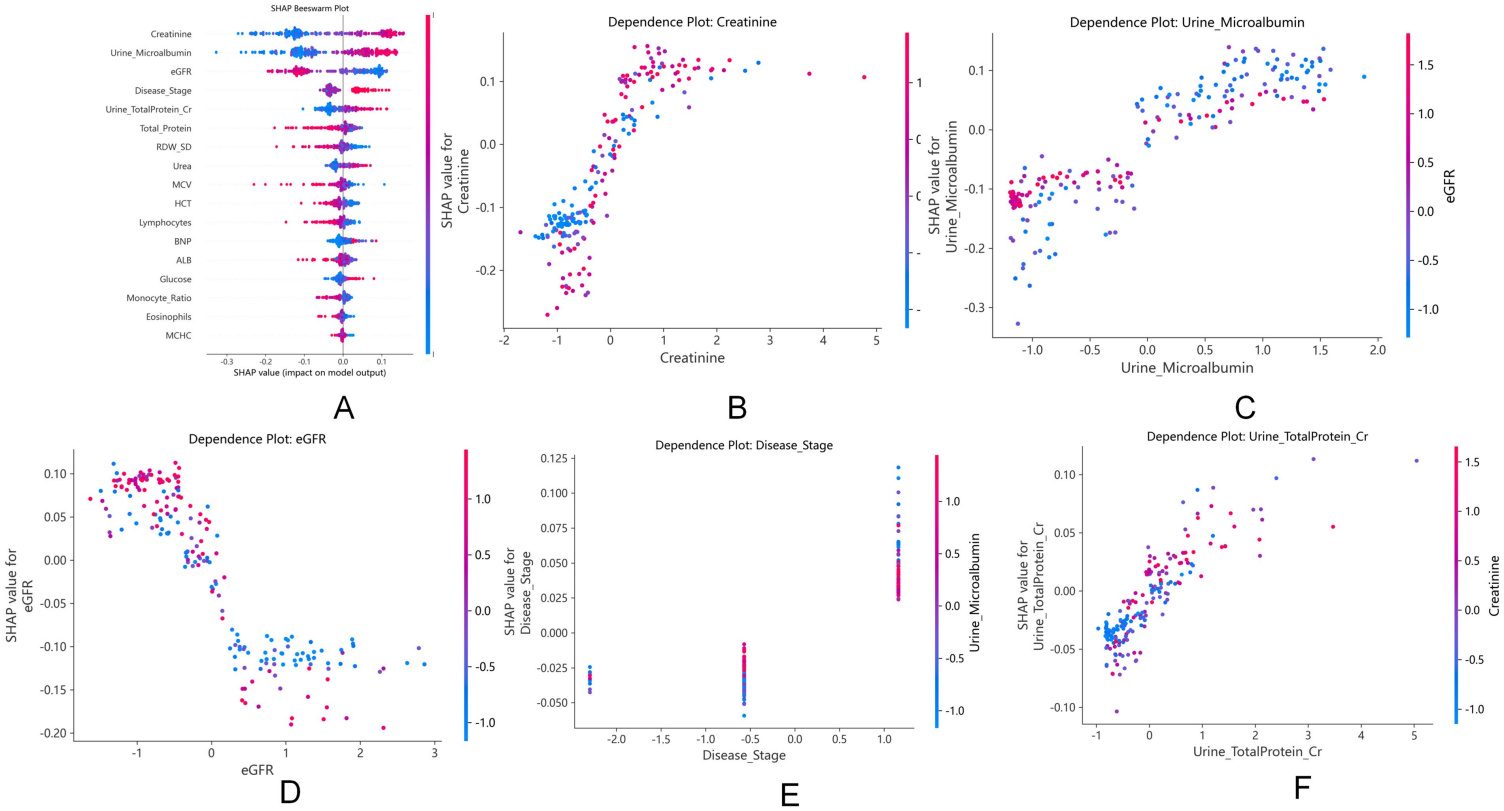
SHAP beeswarm and dependence plots analyzing the effects and interactions of key features on dialysis risk. **(A-F)** represent SHAP beeswarm plot and feature dependence plots respectively, reflecting the effect direction, monotonic correlation and interaction of each predictive feature.

SHAP analysis identified serum creatinine, urine microalbumin, and eGFR as the top three contributors to dialysis risk (Figure 7). As shown in Figure 8A, higher values of serum creatinine, urine microalbumin, CKD disease stage, and urine total protein-to-creatinine ratio increased dialysis risk, while elevated eGFR, total serum protein, RDW_SD, and HCT were protective. Meanwhile, serum creatinine (Figure 8B) and urine total protein-to-creatinine ratio (Figure 8F) presented monotonic positive correlations with dialysis risk, whereas eGFR (Figure 8D) showed a monotonic negative correlation. Furthermore, significant interaction effects were observed between urine microalbumin and eGFR (Figure 8C), as well as between disease stage and albuminuria (Figure 8E), suggesting a synergistic relationship in elevating dialysis risk.

**Figure 9:**
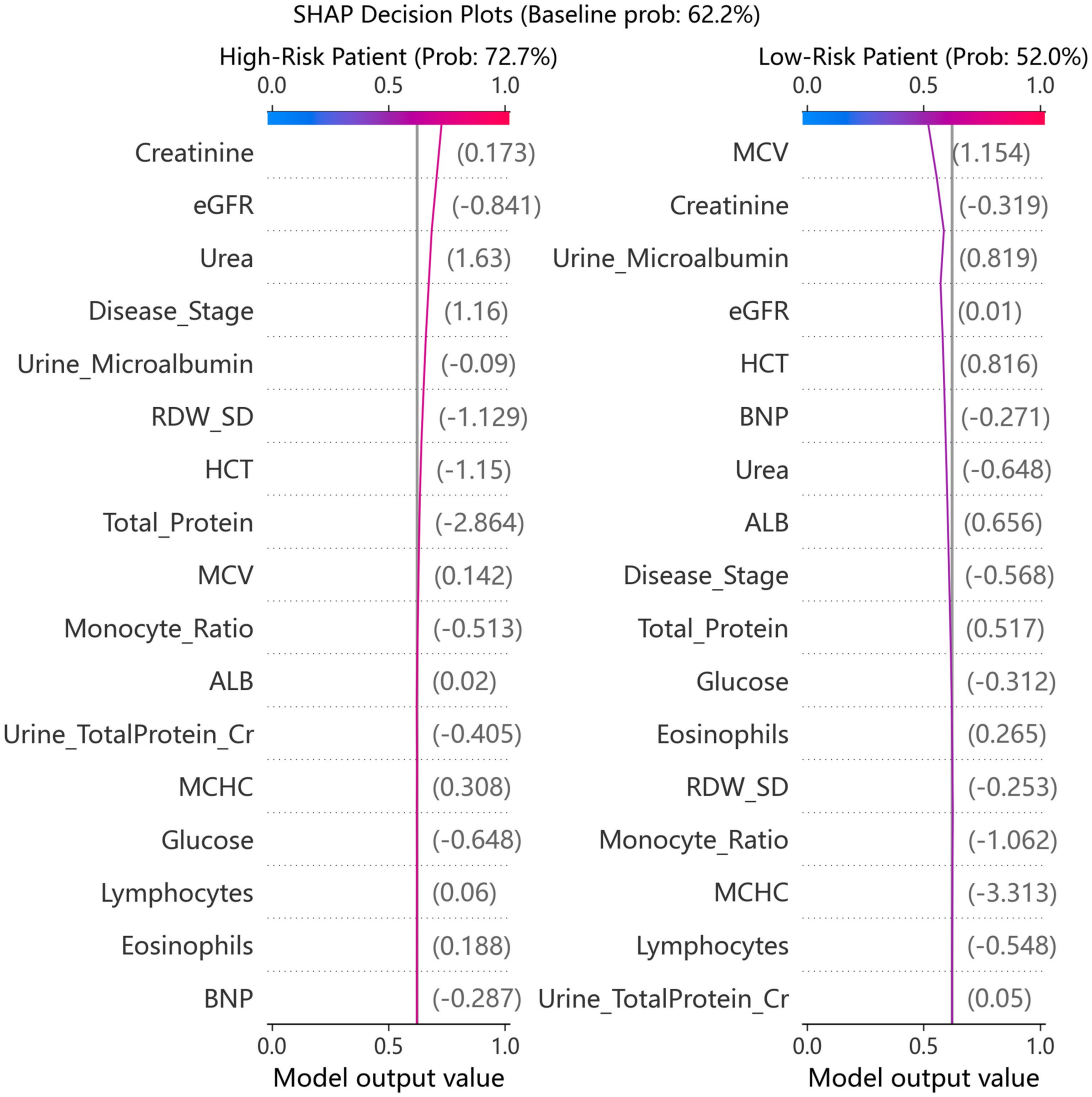
SHAP decision plots for representative individual predictions.

## 4 Discussion

In this study, we developed and internally validated an interpretable machine learning model for predicting dialysis initiation in patients with stage 3–4 chronic kidney disease (CKD), using a parsimonious set of routinely available clinical features. After rigorous correction for data leakage, the Random Forest model maintained excellent discrimination with a mean AUC of 0.988 (95% CI: 0.974–1.003), accuracy of 0.965, and recall of 0.970. Temporal validation on an independent 2024 cohort confirmed the model’s robustness (AUC = 1.000, recall = 1.000), and subgroup analysis demonstrated consistent performance across sex, age, and diabetes strata. SHAP analysis revealed that creatinine, urine microalbumin, and eGFR were the strongest predictors, while also uncovering clinically plausible interactions between proteinuria and erythropoietic dysfunction.

### 4.1 Comparison with prior studies

Our results align with, yet significantly extend, prior work on CKD progression modeling. Earlier studies have consistently identified eGFR decline and proteinuria as key predictors of renal replacement therapy (RRT) initiation (19). However, most existing models either rely on longitudinal eGFR trajectories—limiting their utility at a single timepoint—or achieve modest discrimination (AUC typically 0.80–0.90) (9, 20–23). In contrast, our approach leverages a single baseline assessment and achieves excellent discrimination across multiple algorithms, including simple logistic regression (AUC = 0.949). This suggests that the predictive power lies not in algorithmic complexity alone, but in the multi-domain feature set encompassing renal function, hematologic indices, and metabolic markers.

Notably, the feature selection frequency analysis (Supplementary Table S3) demonstrated that 24 features were selected in all 10 cross-validation folds, indicating robust predictive value. Among these, creatinine emerged as the top predictor, surpassing even urine microalbumin. This finding underscores the central role of muscle-mass-independent filtration markers in advanced CKD, where creatinine may better reflect the cumulative burden of kidney dysfunction (24). The high selection frequency of hematologic parameters (HCT, HGB, MCV, RDW_SD) and metabolic markers (BNP, LDL_C, Glucose) reinforces the concept of CKD as a multisystem disorder (25, 26).

### 4.2 Incremental value beyond traditional predictors

A key concern raised in the peer review was the potential tautological prediction using disease stage alone. To address this, we performed two complementary analyses. First, stratified analysis showed that the model maintained high discrimination within individual CKD stages (Stage 3 AUC = 1.000; Stage 4 AUC = 1.000), confirming its ability to differentiate risk even among patients with similar eGFR levels. Second, baseline model comparison revealed that the full model significantly outperformed a simplified model containing only eGFR and urine microalbumin (AUC: 0.988 ± 0.021 vs. 0.966 ± 0.041; paired t-test *p* = 0.059), with the full model achieving higher AUC in 9 out of 10 folds (Supplementary Table S5). These results demonstrate that the additional hematologic and metabolic markers provide meaningful incremental predictive information beyond traditional risk factor.

### 4.3 Temporal stability and generalizability

The temporal validation performed on the 2024 cohort (30% hold-out) yielded perfect discrimination (AUC = 1.000) with only one misclassified patient, providing strong evidence that the model generalizes well to future patients from the same center. While this does not replace external validation in independent multicenter cohorts, it mitigates concerns about overfitting and temporal shifts in clinical practice. The perfect recall (1.000) in temporal validation is particularly noteworthy, as it ensures that no high-risk patients were missed—a critical requirement for clinical deployment where false negatives carry severe consequences. This emphasis on early detection is consistent with the growing understanding of CKD as a multisystem disorder in which complications such as renal anemia can accelerate disease progression (27).

### 4.4 Subgroup analysis and fairness

Consistent with TRIPOD+AI guidelines (15), we assessed model fairness across key subgroups. The model demonstrated uniformly excellent performance across sex, age, and diabetes strata (all AUCs = 1.000), with Brier scores ranging from 0.013 to 0.037. Notably, for Stage 2 patients (*n* = 22, no events), the model correctly assigned very low predicted probabilities (Brier score = 0.001), confirming its ability to identify low-risk individuals even in the absence of training examples. However, the absence of events in Stage 2 precludes reliable application of the model to this population, and we have therefore revised the scope to focus on Stage 3–4 CKD.

### 4.5 SHAP interpretability and clinical insights

SHAP analysis provided granular, patient-level interpretability that enhances clinical trust. The identification of creatinine, urine microalbumin, and eGFR as top predictors aligns with established nephrology knowledge (1, 4). More intriguingly, we observed that the risk conferred by albuminuria varied with eGFR levels (Figure 8C), and the effect of disease stage was modulated by albuminuria (Figure 8E). These interactions suggest a synergistic relationship between proteinuric injury and erythropoietic dysfunction, resonating with emerging concepts of “renal anemia” as both a consequence and accelerator of CKD progression (25, 26, 28). The protective effects of higher total protein, RDW_SD, and HCT further support the role of nutritional and hematologic status in modulating dialysis risk (24, 29).

### 4.6 Limitations

Several limitations warrant consideration. First, our cohort was derived from a single tertiary center in China, which may limit generalizability to primary care settings or populations with different ethnic and socioeconomic profiles. The high event rate (34.5%) reflects referral bias inherent to tertiary care, and the model may not perform as well in community-based cohorts with lower dialysis incidence. Second, Stage 2 patients were underrepresented (*n* = 22) with no events, preventing reliable model application to early-stage CKD. We have revised the title and scope accordingly to focus on Stage 3–4. Third, due to the retrospective design, we could not accurately record follow-up time for patients who did not initiate dialysis; all outcomes were ascertained as of December 31, 2024, and the prediction horizon was estimated as approximately 12 months, Future prospective studies with complete longitudinal data are needed to define precise prediction windows and enable time-to-event analysis.

Fourth, although temporal validation was performed, external validation in independent multicenter cohorts remains essential before clinical deployment. Variations in dialysis initiation practices, CKD etiology, and healthcare systems across countries could significantly impact model performance. Fifth, our study was not powered to assess the incremental value of novel biomarkers (e.g., FGF23, suPAR); future iterations could integrate such markers to refine prediction in borderline-risk patients.

Finally, the perfect AUC (1.000) in temporal validation, while encouraging, should be interpreted with caution. This may partly reflect the deterministic nature of dialysis initiation in patients with very low eGFR and heavy proteinuria—well-established clinical triggers. The relatively small test set (*n* = 120) may also have contributed to optimistic performance estimates. Reassuringly, the model’s excellent calibration (Brier score = 0.031) and the consistency across subgroups suggest that the findings are robust.

### 4.7 Clinical implications and future directions

Despite these limitations, our model offers a practical tool for early risk stratification using only routine laboratory tests. Its high recall (0.97–1.00) ensures that few high-risk patients are missed, while the SHAP framework provides transparent, individualized explanations to support shared decision-making. Integration into electronic health record systems could enable automated risk alerts during routine clinic visits, prompting timely interventions such as SGLT2 inhibitor initiation, intensified blood pressure control, or expedited nephrology referral (5, 19).

Future research should focus on prospective multicenter validation to assess generalizability across diverse populations and healthcare settings. Randomized implementation trials are needed to determine whether model-guided management improves hard outcomes such as time to dialysis, hospitalization rates, and patient quality of life. Additionally, incorporating dynamic longitudinal data (e.g., eGFR slope, albuminuria trajectory) could further enhance predictive accuracy and enable real-time risk updating.

## 5 Conclusion

In conclusion, we developed and internally validated an interpretable machine learning model that accurately predicts 12-month dialysis risk in patients with stage 3–4 CKD using only routinely collected clinical variables. The model integrates renal, hematologic, and metabolic domains, and SHAP analysis revealed biologically plausible interactions—particularly between proteinuria and erythropoietic dysfunction. With excellent discrimination, calibration, and fairness across subgroups, this approach offers a practical step toward precision nephrology using existing clinical data. External validation and implementation studies are now warranted to translate these findings into improved patient outcomes.

## Data Availability

The data analyzed in this study is subject to the following licenses/restrictions: Deidentified participant data and code used for model development and validation are available upon reasonable request to qualified researchers for purposes of replicating procedures or reproducing results. Requests should be submitted to the corresponding author (3128557854@qq.com) and will be reviewed by the Institutional Data Access Committee. Data use agreements may be required. The data are not publicly available due to privacy and ethical restrictions under The Central Hospital of Wuhan regulations. Requests to access these datasets should be directed to 312855784@qq.com.
