# Supplementary material for: Development and internal validation of an interpretable machine learning model for predicting dialysis risk in patients with stage 3–4 chronic kidney disease
Source: Front Public Health. 2026 Apr 2;14:1782951. doi: 10.3389/fpubh.2026.1782951 (PMC13083080; doi:10.3389/fpubh.2026.1782951)
Supplement: Supplementary file 1 [file Table_1.DOCX]

**Supplementary Table S1. Missing Ratio of Candidate Features**

| **Feature** | **Missing_Ratio(%)** |
| --- | --- |
| N_MID_Osteocalcin | 24.5 |
| B_Collagen | 23.5 |
| Total_25OH_Vitamin_D | 23.0 |
| Urine_TotalProtein_Cr | 23.0 |
| Urine_Total_Protein | 23.0 |
| Urine_Microalbumin | 21.5 |
| UACR | 21.5 |
| TSH | 19.5 |
| BNP | 17.5 |
| PTH | 17.0 |
| Urine_SG | 3.0 |
| HDL_C | 2.5 |
| LDL_C | 2.5 |
| Total_Cholesterol | 2.0 |
| D_Dimer | 1.0 |
| APTT | 0.5 |
| Creatinine | 0.5 |
| Fibrinogen | 0.5 |
| PT_INR | 0.5 |
| PT | 0.5 |
| Hyperuricemia | 0.2 |
| Gender | 0.0 |
| Smoking | 0.0 |
| Marital_Status | 0.0 |
| Age | 0.0 |
| Diabetes | 0.0 |
| CHD | 0.0 |
| Hypertension | 0.0 |
| Alcohol | 0.0 |
| Disease_Stage | 0.0 |
| Cerebral_Infarction | 0.0 |
| Gout | 0.0 |
| Hyperlipidemia | 0.0 |
| HCT | 0.0 |
| Lymphocytes | 0.0 |
| Neutrophil_Ratio | 0.0 |
| Neutrophils | 0.0 |
| HGB | 0.0 |
| RDW_SD | 0.0 |
| PLT | 0.0 |
| Dialysis | 0.0 |
| WBC | 0.0 |
| MCHC | 0.0 |
| RBC | 0.0 |
| RDW_CV | 0.0 |
| MPV | 0.0 |
| Basophil_Ratio | 0.0 |
| PDW | 0.0 |
| Eosinophil_Ratio | 0.0 |
| Basophils | 0.0 |
| Monocyte_Ratio | 0.0 |
| Eosinophils | 0.0 |
| Lymphocyte_Ratio | 0.0 |
| Monocytes | 0.0 |
| MCV | 0.0 |
| MCH | 0.0 |
| P_LCR | 0.0 |
| PCT | 0.0 |
| Glucose | 0.0 |
| UA | 0.0 |
| Urea | 0.0 |
| DBIL | 0.0 |
| TBIL | 0.0 |
| A_G_Ratio | 0.0 |
| GLB | 0.0 |
| Total_Protein | 0.0 |
| AST | 0.0 |
| ALT | 0.0 |
| eGFR | 0.0 |
| P | 0.0 |
| Ca | 0.0 |
| Cl | 0.0 |
| Na | 0.0 |
| K | 0.0 |
| ALB | 0.0 |
| GGT | 0.0 |
| IBIL | 0.0 |
