# Supplementary material for: Development and internal validation of an interpretable machine learning model for predicting dialysis risk in patients with stage 3–4 chronic kidney disease
Source: Front Public Health. 2026 Apr 2;14:1782951. doi: 10.3389/fpubh.2026.1782951 (PMC13083080; doi:10.3389/fpubh.2026.1782951)
Supplement: Supplementary file 2 [file Table_2.DOCX]

Supplementary Table S2. Hyperparameters of Machine Learning Models

| **Model** | **Parameters** |
| --- | --- |
| Logistic Regression | max_iter=1000; class_weight=balanced; random_state=42 |
| Naive Bayes | default=GaussianNB() |
| Decision Tree | max_depth=5; class_weight=balanced; random_state=42 |
| Random Forest | class_weight=balanced; max_depth=8; min_samples_split=5; n_estimators=100 |
| XGBoost | learning_rate=0.1; max_depth=7; n_estimators=50; subsample=0.8 |
| KNN | n_neighbors=5 |
| SVM | kernel=rbf; probability=True; class_weight=balanced; random_state=42; calibration=CalibratedClassifierCV |
| ANN | hidden_layer_sizes=(64, 32); max_iter=1000; random_state=42 |
| Soft Voting | voting=soft; estimators=all 8 base models |
| Weighted Voting | voting=soft; weights=proportional to each model's AUC |
