# Supplementary material for: Development and internal validation of an interpretable machine learning model for predicting dialysis risk in patients with stage 3–4 chronic kidney disease
Source: Front Public Health. 2026 Apr 2;14:1782951. doi: 10.3389/fpubh.2026.1782951 (PMC13083080; doi:10.3389/fpubh.2026.1782951)
Supplement: Supplementary file 3 [file Table_3.DOCX]

Supplementary Table S3. Feature Selection Frequency Across 10-Fold Cross-Validation

| **Feature** | **Selected_Folds** |
| --- | --- |
| Marital_Status | 10 |
| Cerebral_Infarction | 10 |
| CHD | 10 |
| Alcohol | 10 |
| Hyperlipidemia | 10 |
| RDW_SD | 10 |
| PLT | 10 |
| HCT | 10 |
| BNP | 10 |
| RBC | 10 |
| PT | 10 |
| Disease_Stage | 10 |
| Na | 10 |
| Urine_TotalProtein_Cr | 10 |
| Urine_Microalbumin | 10 |
| LDL_C | 10 |
| Creatinine | 10 |
| Glucose | 10 |
| Urea | 10 |
| HGB | 10 |
| Lymphocyte_Ratio | 10 |
| Eosinophils | 10 |
| Monocyte_Ratio | 10 |
| Lymphocytes | 10 |
| MCV | 10 |
| Fibrinogen | 9 |
| Total_Protein | 9 |
| UA | 9 |
| Ca | 8 |
| eGFR | 8 |
| ALT | 8 |
| K | 8 |
| Age | 8 |
| Hyperuricemia | 7 |
| Hypertension | 7 |
| ALB | 7 |
| Urine_SG | 7 |
| PT_INR | 7 |
| Eosinophil_Ratio | 7 |
| Basophil_Ratio | 7 |
| IBIL | 6 |
| DBIL | 6 |
| TSH | 6 |
| Diabetes | 6 |
| B_Collagen | 6 |
| Total_25OH_Vitamin_D | 6 |
| Smoking | 5 |
| Gout | 5 |
| UACR | 5 |
| PCT | 5 |
| Cl | 5 |
| Urine_Total_Protein | 5 |
| PDW | 5 |
| MCHC | 5 |
| RDW_CV | 5 |
| APTT | 5 |
| Monocytes | 5 |
| P_LCR | 5 |
| AST | 5 |
| Total_Cholesterol | 5 |
| D_Dimer | 4 |
| GGT | 4 |
| Gender | 3 |
| GLB | 3 |
| P | 3 |
| WBC | 3 |
| Neutrophil_Ratio | 3 |
| MCH | 3 |
| HDL_C | 3 |
| A_G_Ratio | 3 |
| N_MID_Osteocalcin | 2 |
| MPV | 2 |
| Neutrophils | 2 |
| PTH | 1 |
| Basophils | 1 |
| TBIL | 1 |
