# Supplementary material for: Development and internal validation of an interpretable machine learning model for predicting dialysis risk in patients with stage 3–4 chronic kidney disease
Source: Front Public Health. 2026 Apr 2;14:1782951. doi: 10.3389/fpubh.2026.1782951 (PMC13083080; doi:10.3389/fpubh.2026.1782951)
Supplement: Supplementary file 4 [file Table_4.DOCX]

Supplementary Table S4. Individual Predictions on the Temporal Validation Set

| **ID** | **y_true** | **y_prob** | **y_pred** |
| --- | --- | --- | --- |
| 1 | 0 | 0.298263384276542 | 0 |
| 2 | 0 | 0.0467857142857143 | 0 |
| 3 | 0 | 0.000000000000000 | 0 |
| 4 | 0 | 0.0399209486166008 | 0 |
| 5 | 1 | 0.878074920647443 | 1 |
| 6 | 1 | 0.824145745558789 | 1 |
| 7 | 0 | 0.283967119028772 | 0 |
| 8 | 1 | 0.859211706168228 | 1 |
| 9 | 1 | 0.856525919732441 | 1 |
| 10 | 1 | 0.960768991282690 | 1 |
| 11 | 0 | 0.233566433566434 | 0 |
| 12 | 0 | 0.0640641711229947 | 0 |
| 13 | 0 | 0.0200000000000000 | 0 |
| 14 | 0 | 0.000000000000000 | 0 |
| 15 | 0 | 0.0800000000000000 | 0 |
| 16 | 1 | 0.944194139194139 | 1 |
| 17 | 0 | 0.212856990622336 | 0 |
| 18 | 1 | 0.687288751597037 | 1 |
| 19 | 0 | 0.000000000000000 | 0 |
| 20 | 0 | 0.458930763819315 | 0 |
| 21 | 1 | 0.727393904722280 | 1 |
| 22 | 1 | 0.674515421356856 | 1 |
| 23 | 0 | 0.00714285714285714 | 0 |
| 24 | 1 | 0.907272256728779 | 1 |
| 25 | 0 | 0.139077378253580 | 0 |
| 26 | 0 | 0.208442554887568 | 0 |
| 27 | 1 | 0.867804455818155 | 1 |
| 28 | 0 | 0.109475524475524 | 0 |
| 29 | 0 | 0.270646056841709 | 0 |
| 30 | 0 | 0.368860785420888 | 0 |
| 31 | 1 | 0.777604477752546 | 1 |
| 32 | 0 | 0.144120553359684 | 0 |
| 33 | 0 | 0.335001840264998 | 0 |
| 34 | 0 | 0.294703334423014 | 0 |
| 35 | 1 | 0.647964192670075 | 1 |
| 36 | 0 | 0.00588235294117647 | 0 |
| 37 | 0 | 0.247274476730998 | 0 |
| 38 | 0 | 0.000000000000000 | 0 |
| 39 | 0 | 0.0340579710144928 | 0 |
| 40 | 0 | 0.000000000000000 | 0 |
| 41 | 0 | 0.000000000000000 | 0 |
| 42 | 1 | 0.919524544083368 | 1 |
| 43 | 1 | 0.618312520812521 | 1 |
| 44 | 0 | 0.000000000000000 | 0 |
| 45 | 1 | 0.944339356295878 | 1 |
| 46 | 1 | 0.875330625300846 | 1 |
| 47 | 0 | 0.365740346609912 | 0 |
| 48 | 0 | 0.177533891547049 | 0 |
| 49 | 0 | 0.000000000000000 | 0 |
| 50 | 1 | 0.734773878082702 | 1 |
| 51 | 1 | 0.758711615914817 | 1 |
| 52 | 0 | 0.105000000000000 | 0 |
| 53 | 1 | 0.937593324549846 | 1 |
| 54 | 0 | 0.434882363009402 | 0 |
| 55 | 0 | 0.306521152760283 | 0 |
| 56 | 0 | 0.195846273291925 | 0 |
| 57 | 0 | 0.000000000000000 | 0 |
| 58 | 1 | 0.756325299414544 | 1 |
| 59 | 0 | 0.0125000000000000 | 0 |
| 60 | 1 | 0.906948051948052 | 1 |
| 61 | 0 | 0.0742857142857143 | 0 |
| 62 | 1 | 0.952419840433539 | 1 |
| 63 | 1 | 0.946126134139833 | 1 |
| 64 | 0 | 0.000000000000000 | 0 |
| 65 | 0 | 0.0258823529411765 | 0 |
| 66 | 0 | 0.000000000000000 | 0 |
| 67 | 0 | 0.000000000000000 | 0 |
| 68 | 0 | 0.0200000000000000 | 0 |
| 69 | 0 | 0.182002997002997 | 0 |
| 70 | 1 | 0.874531233046578 | 1 |
| 71 | 1 | 1.000000000000000 | 1 |
| 72 | 0 | 0.146813186813187 | 0 |
| 73 | 1 | 0.892364532019704 | 1 |
| 74 | 0 | 0.000000000000000 | 0 |
| 75 | 0 | 0.159554093567251 | 0 |
| 76 | 0 | 0.187886455649614 | 0 |
| 77 | 0 | 0.000000000000000 | 0 |
| 78 | 0 | 0.0267857142857143 | 0 |
| 79 | 1 | 0.747129059133535 | 1 |
| 80 | 1 | 0.985757575757576 | 1 |
| 81 | 1 | 0.665015186575289 | 1 |
| 82 | 0 | 0.379356776178758 | 0 |
| 83 | 0 | 0.0373913043478261 | 0 |
| 84 | 0 | 0.000000000000000 | 0 |
| 85 | 0 | 0.0250000000000000 | 0 |
| 86 | 0 | 0.0843189163777400 | 0 |
| 87 | 0 | 0.0815909090909091 | 0 |
| 88 | 0 | 0.000000000000000 | 0 |
| 89 | 0 | 0.0258823529411765 | 0 |
| 90 | 0 | 0.178720921695077 | 0 |
| 91 | 0 | 0.580957471461356 | 1 |
| 92 | 0 | 0.337751590514748 | 0 |
| 93 | 0 | 0.285108903245092 | 0 |
| 94 | 0 | 0.0925467914438503 | 0 |
| 95 | 0 | 0.0796428571428571 | 0 |
| 96 | 1 | 0.959178082191781 | 1 |
| 97 | 0 | 0.000000000000000 | 0 |
| 98 | 0 | 0.0333333333333333 | 0 |
| 99 | 0 | 0.0200000000000000 | 0 |
| 100 | 1 | 0.945741758241758 | 1 |
| 101 | 0 | 0.000000000000000 | 0 |
| 102 | 0 | 0.0200000000000000 | 0 |
| 103 | 0 | 0.00588235294117647 | 0 |
| 104 | 0 | 0.0558766233766234 | 0 |
| 105 | 0 | 0.000000000000000 | 0 |
| 106 | 0 | 0.000000000000000 | 0 |
| 107 | 0 | 0.000000000000000 | 0 |
| 108 | 0 | 0.235348484848485 | 0 |
| 109 | 0 | 0.000000000000000 | 0 |
| 110 | 1 | 0.739917017169846 | 1 |
| 111 | 0 | 0.000000000000000 | 0 |
| 112 | 0 | 0.0587700534759358 | 0 |
| 113 | 0 | 0.0400000000000000 | 0 |
| 114 | 0 | 0.00714285714285714 | 0 |
| 115 | 0 | 0.0753846153846154 | 0 |
| 116 | 0 | 0.0400000000000000 | 0 |
| 117 | 0 | 0.0284989858012170 | 0 |
| 118 | 0 | 0.0490909090909091 | 0 |
| 119 | 0 | 0.000000000000000 | 0 |
| 120 | 0 | 0.0551054018445323 | 0 |
