# Supplementary material for: Development and internal validation of an interpretable machine learning model for predicting dialysis risk in patients with stage 3–4 chronic kidney disease
Source: Front Public Health. 2026 Apr 2;14:1782951. doi: 10.3389/fpubh.2026.1782951 (PMC13083080; doi:10.3389/fpubh.2026.1782951)
Supplement: Supplementary file 5 [file Table_5.DOCX]

Supplementary Table S5. AUC Performance in Each Fold of Cross-Validation

| **Fold** | **AUC_Full** | **AUC_Baseline** |
| --- | --- | --- |
| 1 | 1.000 | 0.995 |
| 2 | 1.000 | 1.000 |
| 3 | 0.984 | 0.887 |
| 4 | 0.995 | 0.984 |
| 5 | 1.000 | 1.000 |
| 6 | 0.940 | 0.920 |
| 7 | 1.000 | 0.992 |
| 8 | 1.000 | 0.989 |
| 9 | 0.983 | 0.977 |
| 10 | 1.000 | 0.920 |
